# Sex and Age Dependent Differences in Extracellular Matrix and Cell Phenotypes in the Mouse Heart

**DOI:** 10.1093/geroni/igaf122.669

**Published:** 2025-12-31

**Authors:** Katarzyna Cieslik

**Affiliations:** Houston Methodist Research Institute, Houston, Texas, United States

## Abstract

Fibrosis is one of the hallmarks of aging, which can lead to increased stiffness of the heart, progressing to diastolic dysfunction and heart failure with preserved ejection fraction. While the general mechanisms leading to fibrosis have been well characterized, there is a knowledge gap in our understanding of the distinct mechanisms leading to fibrosis in a male and female aging heart. Mass spectrometry was used to analyze the proteome of decellularized hearts and sorted fibroblasts. As expected, proteome from the old hearts (both sexes) was enriched for various extracellular matrix proteins, mainly involved in collagen fibril assembly. Fibroblasts isolated from the old hearts expressed higher levels of enzymes necessary for collagen processing and crosslinking. Fibroblasts from the old male hearts (as opposed to those from the old female heart) expressed several complement components and other proteins involved in immune responses. Some of the proteins/surface receptors expressed by the fibroblasts in the old hearts are usually expressed by cancer-associated fibroblasts, suggesting pathway similarities between cancer and aging, which have not been investigated in depth in the heart. Finally, Gli1, a transcription factor that plays an important role in cancer progression, is also involved in collagen synthesis in the aging heart and targeting Gli1 expression reduces collagen expression. We also discovered sex differences in mechanisms leading to collagen synthesis and secretion corresponding to tissue stiffness. In conclusion, both sexes present proteome differences, suggesting the activation of distinct pathways that should be targeted to reduce fibrosis more effectively.

